# An Investigation of the *BRCA2* Met1915Thr Polymorphism in Azerbaijani Breast Cancer Patients

**DOI:** 10.3390/medsci13030103

**Published:** 2025-07-31

**Authors:** Zumrud Safarzade, Bayram Bayramov, Nigar Mehdiyeva, Hagigat Valiyeva, Gunay Ahmadova, Rena Kerimova, Qamar Qurbanova, Orkhan Isayev, Adil Allahverdiyev

**Affiliations:** 1 Laboratory of Human Genetics, Genetic Resources Institute of Ministry of Science and Education, Baku AZ1106, Azerbaijan; 2 Department of Natural Sciences, Western Caspian University, Ahmad Rajabli St., 17A, Baku AZ1072, Azerbaijan; 3 Ellab Diagnostic, Department of Genetics, 44 Ashiq Molla Juma St., Baku AZ1025, Azerbaijan; 4 Department of Oncology, Oncological Clinic, Azerbaijan Medical University, Baku AZ1022, Azerbaijan; ladymed77@hotmail.com (N.M.); doctorhagigat1@gmail.com (H.V.); gunayahmadova1982@gmail.com (G.A.); 5 Department of Medical Biology and Genetics, Azerbaijan Medical University, Baku AZ1022, Azerbaijan; rkerimova@amu.edu.az; 6 Molecular Genetics and Genomics Department, Genetic Resources Institute of Ministry of Science and Education, Baku AZ1106, Azerbaijan; gamargurbanova91@gmail.com; 7 Department of Histology, Embryology and Cytology, Azerbaijan Medical University, Baku AZ1022, Azerbaijan; isayev.orkhan@yahoo.com; 8 The V. Y. Akhundov Scientific Research Medical Preventive Institute, Baku AZ1065, Azerbaijan; adilmoglu@gmail.com; 9 Department of Biotechnology, Baku Engineering University, Baku AZ0101, Azerbaijan

**Keywords:** breast cancer, *BRCA2*, Met1915Thr, polymorphism, PCR-RFLP

## Abstract

**Background/Objectives:** Genetic polymorphisms in the *BRCA2* gene have been implicated in breast cancer susceptibility. While numerous studies have investigated this polymorphism, its precise role in breast cancer development remains unclear. Furthermore, to the best of our knowledge, no related studies have been conducted in Azerbaijan. The aim of this study was to determine the distribution of the *BRCA2* Met1915Thr polymorphism (rs4987117) in the Azerbaijani population and to evaluate its potential association with breast cancer risk. **Methods:** A total of 144 breast cancer patients and 152 healthy controls were recruited from the Oncology Clinic of Azerbaijan Medical University between 2021 and 2024. The Met1915Thr polymorphism was genotyped using PCR-RFLP and visualized on a 2% agarose gel. **Results:** A statistically significant association with increased breast cancer susceptibility was observed for the heterozygous Met/Thr genotype (OR = 1.83, 95%CI = 1.08–3.11, *p* = 0.02), the Thr allele (OR = 1.57, 95%CI = 1.12–2.20, *p* = 0.008), and under the dominant inheritance model (OR = 1.83, 95%CI = 1.15–2.90, *p* = 0.01). Notably, this association was more evident among individuals aged over 58 years, in whom the Met/Thr genotype conferred a significantly elevated risk (OR = 2.35, 95%CI = 1.17–4.73, *p* = 0.02). **Conclusions:** The *BRCA2* Met1915Thr polymorphism is associated with an increased risk of breast cancer in the Azerbaijani population. These findings suggest a potential role of this polymorphism in breast cancer susceptibility and highlight the need for further studies in larger cohorts to validate these associations.

## 1. Introduction

Breast cancer is the most frequently diagnosed malignancy and the leading cause of cancer-related mortality among women worldwide, accounting for more than two million new cases and approximately 650,000 deaths each year [1,2,3]. Although most cases are sporadic, about 5–10% are hereditary and attributable to germline alterations in high-penetrance genes, particularly *BRCA1* and *BRCA2* [2,4].

These genes play a crucial role in DNA repair and maintaining the genetic stability of cells. Mutations in *BRCA2* are associated with an increased risk of developing breast cancer and other oncological diseases. Mutations in *BRCA1* and *BRCA2* account for roughly 20–50% of hereditary breast-cancer cases and significantly increase women’s lifetime risk of both breast and ovarian carcinomas [2,5].

A wide spectrum of genetic alterations in the *BRCA1* and *BRCA2* genes has been linked to hereditary breast cancer [6]. While the effects of clearly pathogenic mutations are well established, researchers have also turned their attention to common polymorphisms in *BRCA2*—genetic variations that may not completely disrupt gene function but could still influence breast cancer development. These polymorphisms, often in the form of missense substitutions, are thought to have more moderate effects and may act in combination with other genetic or environmental factors. Variants such as *Asn372His*, *Asp1420Tyr*, and *Met1915Thr* have been examined in large association studies and meta-analyses, though findings remain inconclusive [7,8,9,10,11,12,13,14]. Still, there is growing interest in understanding how these common variants might influence *BRCA2* function, modify tumor behavior, or shift the age of onset. Studying such polymorphisms—especially in genetically underrepresented populations—may offer valuable insight into the more complex and nuanced aspects of hereditary breast cancer risk [15,16].

The Met1915Thr (rs4987117) variant involves a single C/T change at nucleotide 6137 in exon 11 of *BRCA2***.** This alteration replaces a methionine with a threonine at codon 1915, right in the fourth BRC repeat—a region that helps *BRCA2* load *RAD51* onto damaged DNA so it can be accurately repaired [16,17]. Computer-based structural models hint that exchanging a bulky, hydrophobic methionine for a smaller, polar threonine could loosen the BRC4 core and reduce its grip on *RAD51*, potentially slowing double-strand-break repair and allowing genomic instability to creep in [18]. Clinically, the variant has been tied to earlier disease onset and higher-grade tumors in Polish, Russian, and Kazakh cohorts [6,19,20,21]. Yet two large meta-analyses found no clear overall impact on breast-cancer risk [6,22]. Such mixed results—possibly due to ethnic differences in allele frequency, linkage patterns, or study size—make it crucial to test the variant in additional populations. This study therefore examines Met1915Thr in Azerbaijani women to see whether it influences breast-cancer susceptibility in this distinct genetic setting.

Although *BRCA2* polymorphisms like *Met1915Thr* have been studied in various populations, no data currently exist for the Azerbaijani population. Considering the potential for genetic differences across populations and the lack of regional evidence, we set out to examine the frequency of this variant in Azerbaijani breast cancer patients and healthy controls, and to explore whether it may be linked to cancer risk. By providing the first data for this genetically understudied population, this study offers new insight into genetic factors that may shape breast cancer susceptibility in Azerbaijan and adds to the growing understanding of how common *BRCA2* variants influence disease across different genetic backgrounds.

## 2. Materials and Methods

### 2.1. Subjects

A total of 144 women diagnosed with primary breast cancer between 2021 and 2024 at the Oncology Clinic of Azerbaijan Medical University were enrolled in this study. Table 1 summarizes the clinical characteristics of female patients with a histopathologically confirmed diagnosis of primary breast cancer. Peripheral blood samples were collected from each participant, and diagnoses were confirmed by histopathological evaluation. Eligible participants were women aged 25 to 75 years who had been newly diagnosed with breast cancer and had not received any prior oncological treatment (chemotherapy, radiotherapy, or surgery) at the time of sample collection. Only patients with complete clinical data—including age, sex, tumor grade, and TNM stage—were included. Patients were excluded if they had a prior history of any malignancy, previous cancer treatment, incomplete clinical or pathological records, or chronic conditions such as HIV, hepatitis B, or hepatitis C. The study population included individuals with varying tumor grades (Grade I: 18.1%, Grade II: 71.5%, Grade III: 10.4%) and clinical stages (Stage I: 9.7%, Stage II: 41.0%, Stage III: 5.7%, Stage IV: 43.6%). The mean age of the patients was 58 ± 5 years (range: 35–81 years). For subgroup analyses, participants were stratified by age using 58 years as the cutoff, which corresponded closely to both the mean and median age of the cohort, allowing for balanced comparisons between younger and older subgroups.

To compare genotype and allele frequencies, 152 age-matched healthy women were recruited as controls. Controls were matched to cases by age (±5 years), ethnicity, and geographic origin. Inclusion criteria for controls included the absence of any personal or family history of breast or other cancers, no known genetic disorders, and no chronic illnesses, including HIV, hepatitis B, or hepatitis C. Individuals with any of these conditions were excluded. Morphological assessment confirmed the absence of malignancy in all control participants.

The study protocol was approved by the Ethics Committee of the Institute of Genetic Resources. Written informed consent was obtained from all participants in accordance with institutional and ethical standards.

### 2.2. Genotyping

Blood samples were collected in tubes containing EDTA-Na (ethylenediaminetetraacetic acid disodium salt) as an anticoagulant. Genomic DNA was manually extracted using the non-enzymatic salting-out method. The *BRCA2* Met1915Thr genotypes were identified using the polymerase chain reaction–restriction fragment length polymorphism (PCR-RFLP) technique [13].

Each 20 µL PCR reaction mixture contained 10 ng of genomic DNA, 1.25 units of Taq DNA polymerase in 1 × PCR buffer (100 mM Tris-HCl, pH 8.3; 500 mM KCl; 11 mM MgCl_2_; 0.1% agarose), 1.5 mM additional MgCl_2_, 50 mM dNTPs, and 250 nM of each primer. PCR amplification was carried out under the following conditions: initial denaturation at 95 °C for 5 min, followed by 30 cycles of denaturation at 95 °C for 30 s, annealing at 55 °C for 30 s, extension at 72 °C for 30 s, and a final extension at 72 °C for 5 min. Reactions were performed using a Bio-Rad C1000 Touch™ Thermal Cycler (Bio-Rad, Hercules, CA, USA).

The *BRCA2* Met1915Thr variant was amplified using the following primers: forward (sense): 5′-TTGCCAAACGAAAATTATGG-3′; reverse (antisense): 5′-AGATTTTCCACTTGCTGTGC-3′. The resulting 304 bp PCR product was digested overnight with 5 units of the SphI restriction enzyme. Digestion yielded two fragments of 209 bp and 95 bp corresponding to the Thr allele, while the Met allele remained uncut. The restriction fragments were visualized on 3% agarose gels stained with ethidium bromide.

To ensure genotyping accuracy, 10% of the study samples were randomly selected for validation through repeat analysis using the PCR-RFLP method, yielding a 100% concordance rate with the original results.

### 2.3. Statistical Analysis

Genotype and allele frequencies among breast cancer patients and healthy controls were compared using the chi-square (χ^2^) test or Fisher’s exact test, as appropriate. For contingency tables exceeding 2 × 2 dimensions, Fisher’s exact test was applied using the Social Science Statistics platform (http://www.socscistatistics.com/tests/chisquare2/Default2.aspx, accessed on 10 April 2025). The association between genetic polymorphisms and breast cancer risk was assessed by calculating odds ratios (ORs) with corresponding 95% confidence intervals (CIs) using binary logistic regression. Analyses were conducted under both dominant and recessive genetic models. All statistical tests were two-tailed, with a significance threshold set at *p* < 0.05. Statistical analyses were performed using SPSS software (version 22.0; IBM Corp., Chicago, IL, USA). Additionally, post hoc power analysis was conducted using G*Power software (version 3.1.9.7; https://www.psychologie.hhu.de/arbeitsgruppen/allgemeine-psychologie-und-arbeitspsychologie/gpower, accessed on 20 April 2025).

The distribution of *BRCA2* Met1915Thr genotypes was assessed for Hardy–Weinberg equilibrium (HWE) using the chi-square test. Significant deviations from HWE were observed in both the patient group (χ^2^ = 9.522, *p* = 0.0086) and the control group (χ^2^ = 21.063, *p* < 0.0001). These deviations are unlikely to result from genotyping errors and may instead reflect population-specific factors or potential biological relevance.

## 3. Results

The age range of the patient group was 35–81 years, while that of the control group was 42–89 years. The mean ages were 58 ± 5 and 59 ± 5 years, respectively. Based on pathological reports, tumor grades among patients were distributed as follows: Grade I—18.1%, Grade II—71.5%, and Grade III—10.4%. Tumor staging revealed 9.7% of cases in Stage T1, 41.0% in T2, 5.7% in T3, and 43.6% in T4 (Table 1). No statistically significant difference was observed between the patient and control groups with respect to age (*p* > 0.05).

As illustrated in Table 2, a comprehensive overview of the distribution of the *BRCA2* Met1915Thr genotypes is provided, categorised according to the status of breast cancer patients and healthy controls. The Met/Thr genotype was observed to be significantly more prevalent in patients than in controls (OR = 1.83, 95%CI = 1.08–3.11, *p* = 0.02). In a similar manner, individuals exhibiting the Thr/Thr genotype demonstrated a considerably elevated risk (OR = 1.82, 95%CI = 0.99–3.34, *p* = 0.05). A statistically significant association was observed between the heterozygous Met/Thr genotype and increased breast cancer risk. In accordance with the dominant model (Met/Thr + Thr/Thr vs. Met/Met), a significant association with breast cancer susceptibility was identified (OR = 1.83, 95%CI = 1.15–2.90, *p* = 0.01). Conversely, no statistically significant association was observed under the recessive model (Thr/Thr vs. Met/Met + Met/Thr; *p* = 0.21). Allelic frequency analysis revealed that the Thr allele was significantly more common in breast cancer patients (41.7%) than in controls (31.2%), indicating a notable association with increased disease risk (OR = 1.57, 95%CI = 1.12–2.20, *p* = 0.008).

In the present study, genotype distributions were compared according to age stratification, with 58 years—the mean age of the cohort—set as the cutoff point (Table 3). In the subset of individuals aged ≤58 years, the heterozygous Met/Thr genotype (31.9%) and the homozygous Thr/Thr genotype (21.7%) were observed at higher frequencies in patients than in controls. However, these differences did not achieve statistical significance (*p* > 0.05). Conversely, among individuals older than 58 years, the Met/Thr genotype was significantly associated with an increase in the risk of breast cancer (OR = 2.35, 95%CI = 1.17–4.73, *p* = 0.02). Although the Thr/Thr genotype demonstrated a similar trend toward elevated risk (OR = 2.11, 95%CI = 0.96–4.65), the association did not reach statistical significance (*p* = 0.06).

The distribution of *BRCA2* Met1915Thr genotypes in breast cancer patients according to tumor grade and stage is summarized in Table 4. The heterozygous Met/Thr genotype was most prevalent in Grade 1 tumors (50%), whereas the homozygous mutant Thr/Thr genotype was more frequently observed in Grade 2 tumors (27.2%). No statistically significant association was found between genotype distribution and tumor grade (*p* = 0.48).

Regarding tumor stage, the Met/Thr genotype was most common in Stage T4 (38.1%), while the mutant Thr/Thr genotype predominated in Stage T3 (37.5%). No statistically significant association was observed between genotype distribution and tumor stage (*p* > 0.05).

## 4. Discussion

The *BRCA2* gene is essential for maintaining genomic integrity through its central role in homologous recombination–mediated DNA repair. Pathogenic variants in *BRCA2* compromise this repair pathway, thereby promoting genomic instability and increasing vulnerability to malignant transformation. The presence of mutations in the *BRCA2* gene has been demonstrated to be a significant predictor of an elevated risk of developing breast and ovarian cancers [23]. Recent studies suggest that the heritability of breast cancer is partially explained by common low-risk variants and a limited number of moderate- to high-penetrance genes. The findings of the GWAS indicate that the known loci account for approximately 18% of the genetic susceptibility, thus supporting a polygenic model of disease risk [24].

A deeper understanding of how SNPs affect *BRCA2* regulation and expression is essential for advancing breast cancer research and may offer valuable diagnostic and prognostic insights. Among these, the *BRCA2* Met1915Thr (rs4987117) variant has drawn particular attention due to its location within the RAD51-binding BRC repeat domain—critical for DNA repair function—and its potential role in modulating cancer risk. While this SNP has been explored in several populations with conflicting results, data from the South Caucasus region, including Azerbaijan, remain absent. Given the genetic distinctiveness of this population and the existing gap in regional data, our study examined the distribution of this polymorphism and its potential association with breast cancer susceptibility in Azerbaijani women.

In this study, we investigated the Met1915Thr polymorphism of the *BRCA2* gene among breast cancer patients in our population. A statistically significant association was identified between the heterozygous Met/Thr genotype and breast cancer risk. The dominant model also demonstrated a significant difference, whereas no association was found under the recessive model. The increased frequency of the Thr allele among patients suggests a potential link to elevated susceptibility. While these results support a possible role of the Met1915Thr variant in breast cancer risk, they should be interpreted cautiously due to the modest effect sizes and the absence of adjustment for potential confounding variables.

Despite the limited number of studies available in the literature regarding this polymorphism, our findings provide preliminary evidence of its potential role in breast cancer susceptibility. Supporting evidence for our findings is provided by a case–control study conducted by Górski et al. in the Polish population, where the *BRCA2* C5972T (Met1915Thr) polymorphism was significantly associated with an increased risk of breast cancer, particularly among individuals carrying the homozygous genotype [19]. In a follow-up study within the same population, the *BRCA2* Met1915Thr (Thr/Thr) and *BRCA2* Met784Val (Met/Met) polymorphisms were reported as potential independent genetic markers for breast cancer susceptibility [13]. Additionally, a subsequent investigation in the Russian population, specifically in the Voronezh region, identified a notable correlation between the rs4987117 variant of the *BRCA2* gene and the risk of hereditary breast cancer. This variant was suggested to possess high diagnostic and predictive value [6]. Moreover, a significantly elevated frequency of several *BRCA2* polymorphisms—including rs55886062, rs3918290, rs12721655, rs4987117, rs2229774, rs11203289, rs137852576, rs11571833, rs80359062, and rs11571746—was observed in patients with breast adenocarcinoma compared to healthy controls. These variants have been proposed as potential genetic markers for identifying women at elevated risk for developing breast cancer [20]. Another study further confirmed a statistically significant association between the *BRCA2* Met1915Thr variant (rs4987117) and increased breast cancer risk [21]. When considered along with the other findings, these results suggest that this polymorphism may have utility as a genetic marker for breast cancer predisposition. They also underscore the importance of integrating genetic screening into risk stratification protocols, particularly in populations with similar genetic backgrounds.

Contrary to our findings, a combined case–control study conducted by Serrano-Fernández and colleagues in populations from Poland and Belarus reported a protective effect of the *BRCA2* missense variant T1915M [25]. This polymorphism exhibited a substantial correlation with a reduced risk of breast cancer, suggesting that its influence on disease susceptibility might vary among different populations or be influenced by additional genetic and environmental factors.

Numerous studies in the existing literature have reported no statistically significant association between the presence of the *BRCA2* Met1915Thr polymorphism and breast cancer risk. Although several missense polymorphisms in the *BRCA1* and *BRCA2* genes (including *BRCA1* Gln356Arg, Pro871Leu, Glu1038Gly, Ser1613Gly, Met1652Ile, and *BRCA2* Asn289His, Asn372His, Asp1420Tyr, Tyr1915Met) have been hypothesized to influence cancer susceptibility, Dombernowsky et al. concluded that the increased risk of breast and/or ovarian cancer observed in hereditary cancer families cannot be explained solely by heterozygosity or homozygosity of any of these nine variants [26]. Similarly, a meta-analysis by Faramarzi et al. found no statistically significant association between the *BRCA2* Met1915Thr (rs4987117) variant and breast cancer risk [22]. Moreover, in a comprehensive analysis of 1,037 functional single-nucleotide polymorphisms (SNPs), Johnson et al. did not identify the rs4987117 variant among the 21 polymorphisms associated with breast cancer susceptibility [16]. Despite studies suggesting a role for the *BRCA2* Met1915Thr polymorphism in breast cancer risk, a considerable number of investigations have failed to confirm a consistent association. This variability may reflect differences in population genetics, sample sizes, or study designs, underscoring the necessity for additional large-scale, well-controlled studies to elucidate the contribution of this variant to breast cancer susceptibility.

Age appears to be an important modifier in the association between the *BRCA2* Met1915Thr polymorphism and breast cancer risk. When stratifying by age, no statistically significant difference in genotype distribution was observed between patients and controls under 58 years of age in our study. Conversely, individuals aged 58 and above carrying the heterozygous Met/Thr genotype demonstrated a significantly increased risk of breast cancer. Notably, these findings differ from a Polish study that reported an elevated risk associated with the heterozygous Met/Thr genotype in individuals younger than 40 years, while the homozygous Thr/Thr genotype was linked to increased risk in those older than 40 [19]. Such inconsistencies highlight the potential impact of genetic backgrounds unique to specific populations and environmental factors, emphasizing the necessity of incorporating age stratification into genetic association studies of breast cancer.

The distribution of genotypes by tumor stage and grade was assessed, and no statistically significant associations were identified. Similarly, Krupa et al. (2009) investigated the association of two *BRCA2* gene polymorphisms, Thr1915Met (rs4987117) and Met784Val (rs11571653), with clinical parameters in sporadic breast cancer patients [2]. Although no significant correlations were found between these polymorphisms and tumor grade or stage, the variant genotypes of both were inversely associated with hormone receptor-positive status [2].

This study provides important preliminary insights into the potential association between the *BRCA2* Met1915Thr polymorphism and breast cancer risk. However, several limitations must be acknowledged when interpreting these findings. Firstly, the relatively small sample size may have limited the statistical power to detect modest associations, especially in subgroup analyses or for rare genotypes. Secondly, the study population was limited to a specific ethnic and geographic group, which may restrict the generalizability of results to other populations. The analysis focused primarily on basic demographic and tumor-related parameters (age, grade, and stage), without incorporating essential clinical and molecular characteristics such as HER2 status, hormone receptor expression (ER, PR), or molecular subtype. Additionally, relevant patient-specific variables—including body mass index (BMI), menopausal status, family history of cancer, and treatment details—were not available. Significant deviation from Hardy–Weinberg equilibrium was observed in both study groups. However, sequencing validation confirmed genotyping accuracy, suggesting that the deviation may be due to population structure or selection bias rather than technical error. The absence of functional assays also limits the ability to draw mechanistic conclusions regarding the biological role of the Met1915Thr variant. Furthermore, the borderline statistical significance and wide confidence intervals observed in some associations suggest that these results should be interpreted with caution. Future studies incorporating larger, more diverse cohorts, comprehensive clinical and molecular profiling, and functional analyses are warranted to confirm and expand upon these findings.

## 5. Conclusions

In conclusion, our findings suggest a modest but statistically significant association between the *BRCA2* Met1915Thr polymorphism and an increased risk of breast cancer. Both the heterozygous Met/Thr genotype and the mutant T allele were more prevalent among patients, supporting the potential role of this variant in breast cancer susceptibility within the Azerbaijani population. These results provide valuable preliminary evidence and emphasize the importance of further large-scale studies to confirm and expand upon the clinical relevance of this association.

## Figures and Tables

**Table 1:** Clinical characteristics of the study groups.

	Patients N = 144 (%)	Controls N = 152 (%)	*p* Value
Age			0.259
Age interval	35–81	42–89	
Mean	58 ± 5	59 ± 5	
Grade			
I	26 (18.1)		
II	103 (71.5)		
III	15 (10.4)		
TNM staging			
I	14 (9.7)		
II	59 (41)		
III	8 (5.7)		
IV	63 (43.6)		

**Table 2:** Frequency of Met1915Thr genotypes of the *BRCA2* Gene in subject groups.

Genotype	Patients N = 144 (%)	Controls N = 152 (%)	OR (95%CI)	*p* Value
Met/Met	58 (40.3)	84 (55.3)	1	-
Met/Thr	52 (36.1)	41 (27)	1.83 (1.08–3.11)	0.02
Thr/Thr	34 (23.6)	27 (17.7)	1.82 (0.99–3.34)	0.05
Dominant Model				
Met/Met	58 (40.3)	84 (55.3)	1	-
Met/Thr + Thr/Thr	86 (59.7)	68 (44.7)	1.83 (1.15–2.90)	0.01
Recessive Model				
Met/Met + Met/Thr	110 (76.4)	125 (82.3)	1	-
Thr/Thr	34 (23.6)	27 (17.7)	1.43 (0.81–2.52)	0.21
Allele				
Met	168 (58.3)	209 (68.8)	1	-
Thr	120 (41.7)	95 (31.2)	1.57 (1.12–2.20)	0.008

**Table 3:** Distribution of *BRCA2* gene Met1915Thr genotypes according to age.

Age ≤ 58	Patients, N = 69 (%)	Controls, N = 51 (%)	OR (95%CI)	*p* Value
Met/Met	32 (46.4)	29 (56.9)	1	-
Met/Thr	22 (31.9)	14 (27.5)	1.42 (0.61–3.29)	0.41
Thr/Thr	15 (21.7)	8 (15.6)	1.69 (0.62–4.59)	0.29
Age > 58	Patients, N = 75 (%)	Controls, N = 101 (%)		
Met/Met	26 (34.7)	55 (54.5)	1	-
Met/Thr	30 (40)	27 (26.7)	2.35 (1.17–4.73)	0.02
Thr/Thr	19 (25.3)	19 (18.8)	2.11 (0.96–4.65)	0.06

**Table 4:** Distribution of *BRCA2* gene Met1915Thr genotypes by tumor grade and stage.

	Met/Met N (%)	Met/Thr N (%)	Thr/Thr N (%)	*p* Value
Tumor grade				0.48
G1	10 (38.5)	13 (50)	3 (11.5)	
G2	42 (40.8)	33 (32)	28 (27.2)	
G3	6 (40)	6 (40)	3 (20)	
Tumor stage				0.88
T1	6 (42.9)	5 (35.7)	3 (21.4)	
T2	25 (42.4)	21 (35.6)	13 (22)	
T3	3 (37.5)	2 (25)	3 (37.5)	
T4	24 (38.1)	24 (38.1)	15 (23.8)	

## Data Availability

All data employed in this research are presented in the tables included in this article.

## Abbreviations

The following abbreviations are used in this manuscript:

ORs	Odds Ratios
CIs	Confidence Intervals
WHO	World Health Organization
Met	Methionine
Thr	Threonine
EDTA-Na	Ethylenediaminetetraacetic Acid Disodium Salt
